# Eriodictyol attenuates arsenic trioxide-induced liver injury by activation of Nrf2

**DOI:** 10.18632/oncotarget.19822

**Published:** 2017-08-02

**Authors:** Guanghong Xie, Xiaolin Meng, Fei Wang, Yuxin Bao, Junyuan Huo

**Affiliations:** ^1^ College of Veterinary Medicine, Jilin University, Changchun 130062, China

**Keywords:** eriodictyol, arsenic trioxide, liver injury, Nrf2

## Abstract

Arsenic, a well-known human carcinogen, has been reported to induce hepatic oxidative stress and hepatic injury. Eriodictyol, a flavonoid found in citrus fruits, has been reported to have antioxidant effects. In this study, we aimed to investigate the protective effects of eriodictyol on arsenic trioxide (As_2_O_3_)-induced liver injury and to clarify the molecular mechanism. Male Wistar rats were administrated 3mg/kg As_2_O_3_ intravenous injection at days 1, 4, 5, and 7. Eriodictyol was given 1 h before or after As_2_O_3_ treatment. The results showed that eriodictyol prevented As_2_O_3_-induced liver reactive oxygen species (ROS) and malonaldehyde (MDA) levels. Eriodictyol abrogated As_2_O_3_-induced decrease of the antioxidant enzymes superoxide dismutase (SOD), glutathione peroxidase (GPX), and catalase (CAT) activity. Eriodictyol also attenuated As_2_O_3_-induced hepatic pathological damage. In addition, eriodictyol promoted the expression of nuclear factor erythroid 2 p45 related factor 2 (Nrf2) and heme oxygenase-1 (HO-1) up-regulated by As_2_O_3_. In conclusion, our results demonstrated that eriodictyol exhibited a protective effect on As_2_O_3_-induced liver injury and the possible mechanism is involved in activating Nrf2 signaling pathway.

## INTRODUCTION

Arsenic, a well known cytotoxic environmental toxicant, is present in soil, drinking water, and food [1]. Arsenic exposure causes various hazardous effects in human including carcinogenesis in lungs, livers, and bladders [2, 3]. Previous studies showed that arsenic-induced oxidative stress is the main reason of arsenic-induced carcinogenesis [4]. Liver is the major target organ of arsenic. Arsenic-induced liver injury is closely associated with oxidative stress and previous reports indicated that antioxidants had therapeutic effects against arsenic-induced liver injury [5, 6]. Nrf2, an important transcription factor, has been demonstrated to play critical roles in cellular defense against oxidative stress [7]. Activating of Nrf2 had the ability to protect against arsenic-induced liver injury [8].

Eriodictyol, a flavonoid found in citrus fruits, has been reported to have anti-inflammatory, anti-apoptotic, and antioxidant effects. Eriodictyol has been reported to prevent early retinal and plasma abnormalities in streptozotocin-induced diabetic rats [9]. Eriodictyol also protected against hydrogen peroxide-induced neurotoxicity in cultured rat pheochromocytoma cells [10]. Furthermore, eriodictyol was found to exhibit antioxidant effect on UV-induced apoptosis in keratinocytes [11]. However, the protective effects and molecular mechanism of eriodictyol on arsenic trioxide-induced liver injury remain unclear. In this study, we sought to determine whether eriodictyol had protective effects on arsenic trioxide-induced liver injury in rats.

## RESULTS

### Effects of eriodictyol on As_2_O_3_-induced liver histopathologic changes

**Figure 1:**
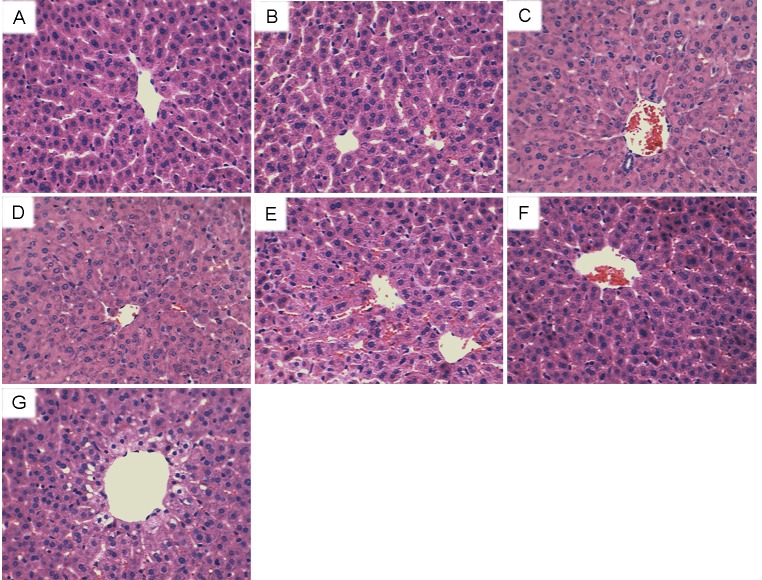
Effects of eriodictyol on As_2_O_3_-induced liver histopathologic changes Representative histological changes of liver obtained from rats of different groups. **(A)**: Control group, **(B)**: eriodictyol (40mg/kg) group, **(C)**: As_2_O_3_ group, **(D)**: eriodictyol (10mg/kg) + As_2_O_3_ group, **(E)**: eriodictyol (20mg/kg) + As_2_O_3_ group, **(F)**: eriodictyol (40 mg/kg) + As_2_O_3_ group, **(G)**: eriodictyol (40 mg/kg) + As_2_O_3_ group (1 h after) (Hematoxylin and eosin staining, magnification 200×).

To investigate the protective effects of eriodictyol on As_2_O_3_-induced liver injury, histopathological changes of liver tissues were tested by H&E staining. As shown in Figure 1, liver histological sections of control group and eriodictyol alone group showed normal structures. Liver histological sections of As_2_O_3_-treated mice showed serious pathological changes, such as extensive areas of portal inflammation, cellular necrosis, and inflammatory cells infiltration. However, As_2_O_3_-induced liver histopathologic changes were markedly ameliorated by treatment of eriodictyol.

### Effects of eriodictyol on ROS and MDA levels in liver tissues

**Figure 2:**
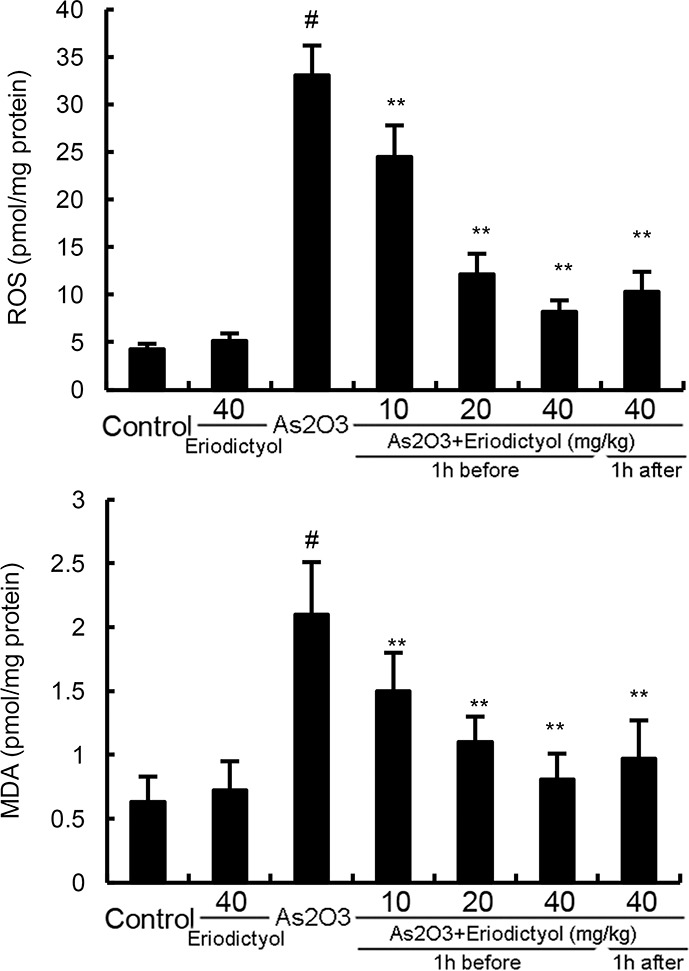
Effects of eriodictyol on As_2_O_3_-induced ROS and MDA levels The values presented are the means ± SEM (n=12 in each group). #p*<0.01 vs.* control group, *p*<0.05* and **p*<0.01 vs.* As_2_O_3_ group.

To investigate the anti-oxidative effects of eriodictyol, the effects of eriodictyol on As_2_O_3_-induced ROS and MDA levels were detected. As shown in Figure 2, treatment of eriodictyol alone did not affect the levels of ROS and MDA. The levels of ROS and MDA increased significantly of As_2_O_3_-treated group than that of the control group. However, in As_2_O_3_ + eriodictyol treated group, the levels of ROS and MDA in liver tissues decreased significantly than that of the As_2_O_3_-treated group.

### Effects of eriodictyol on SOD, GPX, and CAT activity in liver tissues

**Figure 3:**
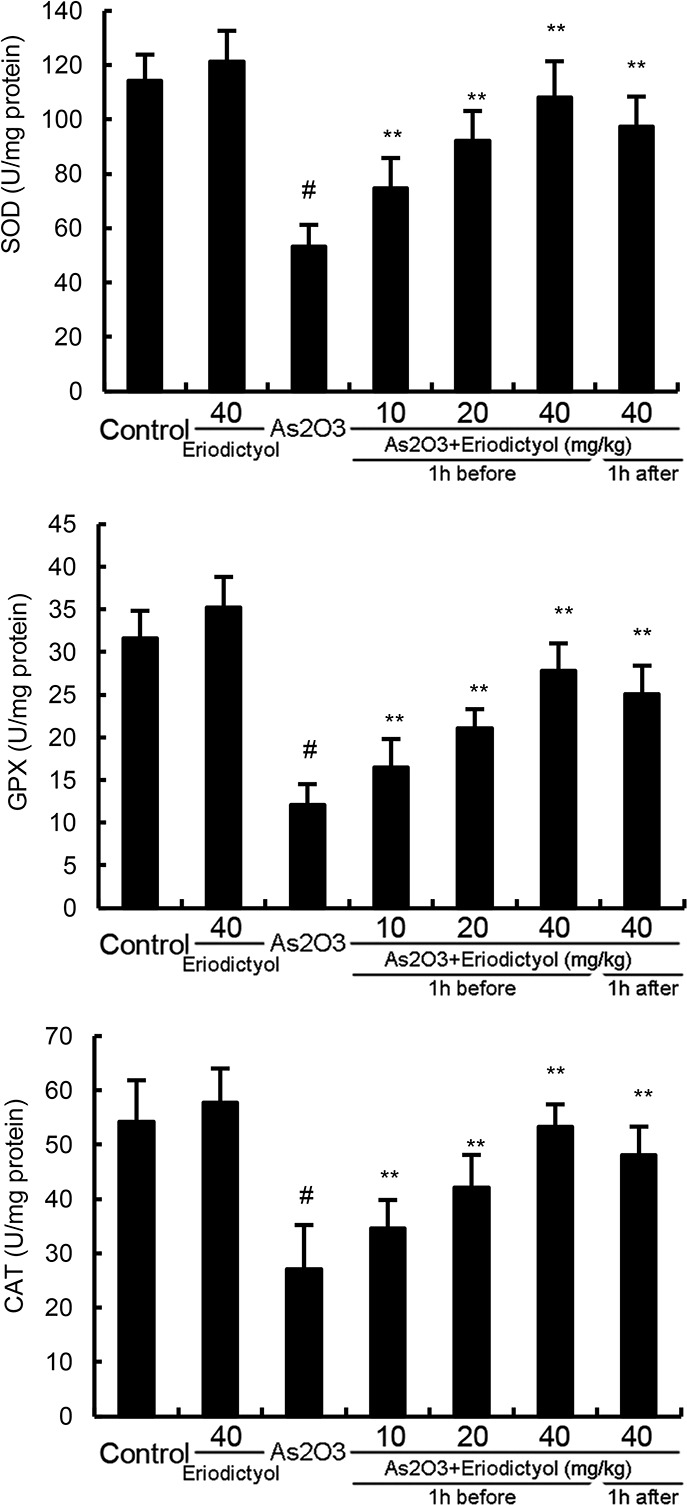
Effects of eriodictyol on As_2_O_3_-induced antioxidant enzymes SOD, GPX, and CAT activity The values presented are the mean ± SEM (n=12 in each group). p#<0.01 vs. control group, p*<0.05, p**<0.01 vs. As_2_O_3_ group.

The effects of eriodictyol on antioxidant enzymes SOD, GPX, and CAT activity were also detected in this study. The results showed that treatment of eriodictyol alone did not affect the activity of SOD, GPX, and CAT. As_2_O_3_ significantly inhibited the activity of antioxidant enzymes SOD, GPX, and CAT. However, the inhibition of SOD, GPX, and CAT activity by As_2_O_3_ was abrogated by eriodictyol (Figure 3).

### Effects of eriodictyol on As_2_O_3_-induced ALT and AST levels in serum

**Figure 4:**
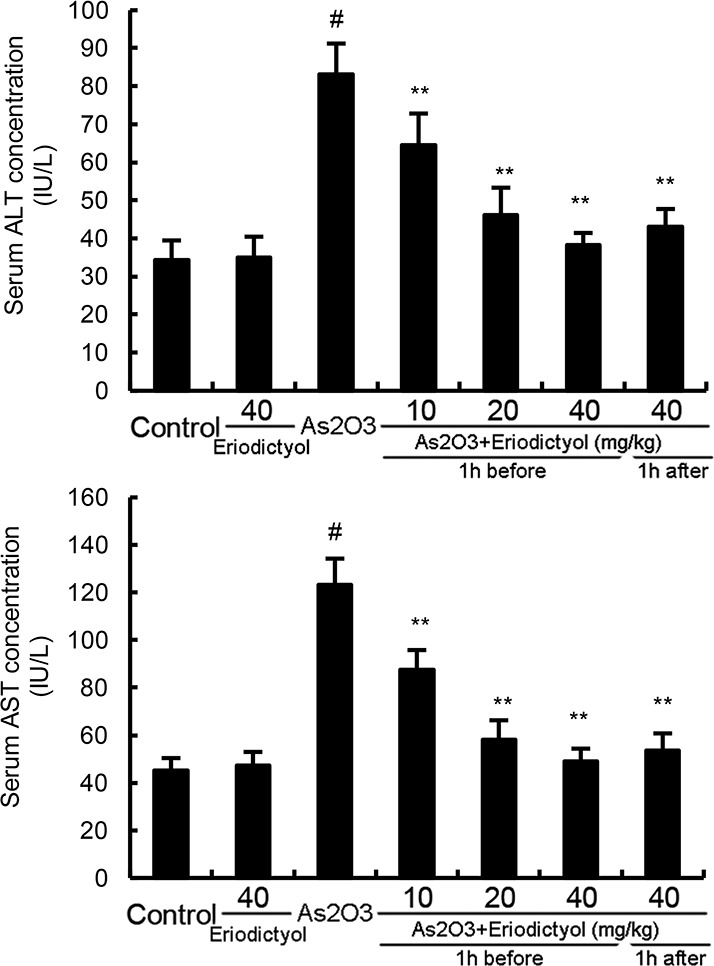
Effects of eriodictyol on As_2_O_3_-induced ALT and AST levels The values presented are the means ± SEM (n=12 in each group). #p*<0.01 vs.* control group, *p*<0.05* and **p*<0.01 vs.* As_2_O_3_ group.

The effects of eriodictyol on As_2_O_3_-induced ALT and AST levels in serum were detected in this study. As shown in Figure 4, treatment of eriodictyol alone did not affect the levels of ALT and AST. The levels of ALT and AST increased significantly of As_2_O_3_-treated group than that of the control group. However, eriodictyol significantly inhibited As_2_O_3_-induced ALT and AST levels.

### Eriodictyol promoted the expression of Nrf2 and HO-1

**Figure 5:**
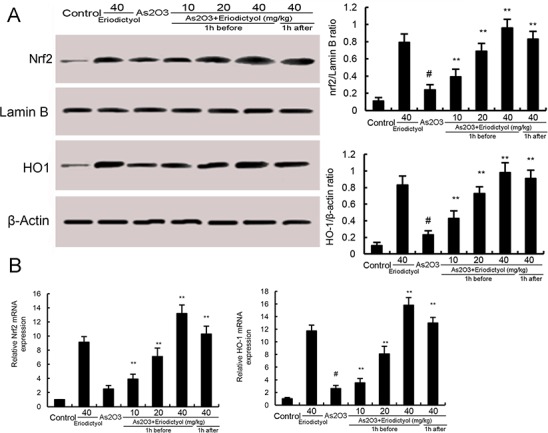
**(A)** Effects of eriodictyol on Nrf2 and HO-1 protein expression. **(B)** Effects of eriodictyol on Nrf2 and HO-1 mRNA expression. The values presented are the means ± SEM (n=12 in each group). #p*<0.01 vs.* control group, *p*<0.05* and **p*<0.01 vs.* As_2_O_3_ group.

To investigate the anti-oxidative mechanism of eriodictyol, the effects of eriodictyol on Nrf2 and HO-1 expression were measured by qRT-PCR and western blot analysis. The results showed that the mRNA and protein expression of Nrf2 and HO-1 were increased by treatment of As_2_O_3_. However, eriodictyol up-regulated the expression of Nrf2 and HO-1 induced by As_2_O_3_ (Figure 5). Furthermore, treatment of eriodictyol alone could drastically increase the cellular Nrf2 and HO-1 expression even without the As_2_O_3_ induction (Figure 5).

## DISCUSSION

Arsenic has been known to induce liver oxidative stress and liver injury. Eriodictyol, a flavonoid found in citrus fruits, has been reported to have antioxidant effect. In this study, we found that eriodictyol attenuated As_2_O_3_-induced oxidant injury in liver tissues by activating Nrf2/HO-1 signaling pathway.

Liver is the major target organ of many toxic chemicals [12]. The underlying mechanism of As_2_O_3_-induced liver injury has not yet completely understood. However, recent studies indicated that oxidative stress induced by As_2_O_3_ was the major reason that associated with liver injury [13, 14]. A large number of studies showed that antioxidants had therapeutic effects against arsenic-induced tissues injury [6, 15]. Eriodictyol has been reported to have antioxidant effect [16]. Thus, we detected the protective effects of eriodictyol on As_2_O_3_-induced liver injury. Our results showed that eriodictyol could attenuate As_2_O_3_-induced pathological changes of liver tissues. Serum ALT and AST were used as biochemical indicator of hepatic injury [17]. In this study, we found that eriodictyol inhibited As_2_O_3_-induced ALT and AST production. Taken together, these results suggested that eriodictyol exhibited protective effects of As_2_O_3_-induced liver injury.

Arsenic exposure exhibits oxidative stress by inducing the production of ROS in liver tissues [18]. In the present study, we found that the production of ROS increased significantly after arsenic exposure. MDA, a significant lipid peroxidation product, increases in oxidative stress [19]. It can be used to monitor the oxidative damage [20]. The levels of MDA in liver tissues increased significantly after arsenic exposure. However, treatment of eriodictyol remarkably suppressed As_2_O_3_-induced MDA and ROS production. Furthermore, the inhibition of SOD, GPX, and CAT activity by As_2_O_3_ was reversed by eriodictyol. These results demonstrated that eriodictyol reduced As_2_O_3_-induced oxidative stress and liver damage. Nrf-2, a basic leucine zipper transcription factor, has been reported to play critical roles in orchestrating cellular antioxidant defenses [21]. Activation of Nrf-2 leads to the expression of HO-1, a cytoprotective enzyme important in heme catabolism [22]. Previous studies showed that Nrf-2 can be used as an effective molecular target to counteract As-induced toxicity [23]. In this study, we found that eriodictyol up-regulated the expression of Nrf2 and HO-1 induced by As_2_O_3_.

In conclusion, our results demonstrated that eriodictyol had a protective effect on As_2_O_3_-induced liver injury. The possible mechanism is involved in activating Nrf2/HO-1 signaling pathway. Eriodictyol has a potential application to treat arsenic-induced toxicity.

## MATERIALS AND METHODS

### Materials

Eriodictyol (purity>98%) was obtained from the Department of Natural Products Chemistry of Shandong University (Jinan, China). As_2_O_3_ parenteral solution was purchased from Harbin Yida Pharmaceutical Company Ltd. (Harbin, China). GPX, SOD, CAT, and MDA kits were purchased from the Jiancheng Bioengineering Institute of Nanjing (Nanjing, China). The Nrf2, HO-1, Lamin B, β-actin, and horseradish peroxidase-conjugated (HRP) secondary antibodies were purchased from Cell Signaling Technology Inc (Beverly, MA).

### Animals

Male Wistar rats (8 week old) were purchased from the Center of Experimental Animals of Jilin University (Changchun, China). All animals were housed in microisolator cages and fed standard laboratory chow and water ad libitum. The environment temperature was 25 ± 2°C and humidity was 55 ± 5%. All animal experiments were performed in accordance with the Health’s Guide for the Care and Use of Laboratory Animals published by the US National Institute of Health.

### Experimental protocol

Eighty-four rats were randomly divided into seven groups: control group, Eriodictyol alone treated group, As_2_O_3_ group, As_2_O_3_ + eriodictyol (10, 20, 40mg/kg, 1 h before) treated groups, and As_2_O_3_ + eriodictyol (40mg/kg, 1 h after) treated group. Male Wistar rats were administrated 3mg/kg As_2_O_3_ intravenous injection at days 1, 4, 5, and 7. Eriodictyol was given by an intraperitoneal injection 1 h before or after As_2_O_3_ treatment. Control rats were treated with equal amount of 0.9% normal saline as a vehicle control. On the 8th day, the rats were killed and the blood samples and livers were collected.

### Histological analysis

To evaluate the changes of liver issues, the livers were obtained and fixed in 10% neutral buffered formalin overnight. The the tissues were dehydrated, paraffin embedded and sliced. The sections were stained with hematoxylin and eosin (H&E) stain. Finally, the pathological changes of liver tissues were observed under a light microscope.

### Measurement of oxidative stress and antioxidant enzymes in liver tissues

Liver tissues were homogenized and the levels of MDA, the antioxidant enzymes GPX, SOD, and CAT in liver tissues were tested by commercial kits (Jiancheng Bioengineering Institute of Nanjing, China) according to the manufacturer’s instructions.

### Measurement of ALT an AST

24 h after the last time of As_2_O_3_ treatment, the blood samples were collected and centrifuged at 3000 g for 8 min to obtain the serum. Serum ALT and AST levels were measured by commercial kits (Jiancheng Bioengineering Institute of Nanjing, China) according to the manufacturer’s instructions.

### Quantitative real-time PCR

TRizol was used to extract the total RNA following the manufacturer’s instructions (Invitrogen, Carlsbad, CA, USA). Then, the RNA samples were reversed transcription to cDNA with the Revert Aid First Strand cDNA Synthesis Kit (MBI Fermentas, Lithuania). Real-time PCR (RT-PCR) was completed on a 7500 real-time PCR system (Applied Biosystems, Carlsbad, CA, USA). The primers were: Nrf2 (F: TTGTAGATGACCATGAGTC, R: TGTCC TGCTGTATGCTGCTT), HO-1 (F: TAAATGCAGTG TTGGCCCC, R: ATGTGCCAGGCATCTCCTT), β-actin (F: GGAGTACGATGAGTCCGGC, R: CGCAGCTCAG TAACAGTCC). Primers were acquired from Sangon Biotech Co. Ltd (Shanghai, China). The reaction conditions of PCR are as follows: 50°C for 2 min and 95°C for 10 min followed by 40 cycles of 95°C for 15 s and 60°C for 1 min. Three replicates of every group were run.

### Western blot analysis

Liver tissues were homogenized in liquid nitrogen and incubated in lysis buffer containing protease and phosphatase inhibitors (Roche, Basel, Switzerland) to obtain protein. The protein concentration was determined through BCA kits. Equal amounts of protein were separated by 12% SDS-PAGE gel and electrotransferred to a nitrocellulose membrane. The membrane was incubated with the primary antibodies Nrf-2 (1: 1000), HO-1 (1: 1000) at 4°C overnight and then probed with HRP-conjugated secondary antibody at room temperature for 2 h. The immunoreactive proteins were detected using an enhanced chemiluminescence Western blotting detection kit.

### Statistical analysis

Data are presented as the mean±SEM. Comparisons between groups were analyzed using one-way ANOVA (Dunnett’s *t*-test) and two tailed Student’s *t*-test. The P< 0.05 or P< 0.01 were considered statistically significant.
